# Editorial: Topical Collection “Ethical and Societal Implications of AgeTech”

**DOI:** 10.1007/s11948-024-00521-0

**Published:** 2024-12-02

**Authors:** Giovanni Rubeis, Andrew Sixsmith

**Affiliations:** 1 Department General Health Studies, Division Biomedical and Public Health Ethics, Karl Landsteiner University of Health Sciences, Dr.-Karl-Dorrek-Straße 30, Krems, 3500 Austria; 2 Department of Gerontology, Simon Fraser University, Burnaby, Canada

**Keywords:** Assistive technologies, Ambient assisted living, Ethics, Gerontology, Robotics, Artificial Intelligence

## Abstract

AgeTech refers to a growing sector that is advancing the use of technologies, such as information and communication technologies (ICTs), mobile technologies, robotics, wearables and smart home systems to enhance the lives of older adults. Although AgeTech can be seen as an opportunity for empowering older people and enhance their overall quality of life, crucial ethical issues have to be addressed. The articles in this topical collection focus on these and other ethical questions, particularly in respect to key emerging technologies of AI and robotics. The overall aim is to explore the multifaceted ethical landscape of emerging AgeTech and to provide frameworks and strategies for ethically-appropriate technologies that support the health, well-being, and quality of life of older adults.

AgeTech refers to a growing sector that is advancing the use of technologies, such as information and communication technologies (ICTs), mobile technologies, robotics, wearables and smart home systems to enhance the lives of older adults. While AgeTech has primarily focused on older people with age-related chronic diseases and disabilities, increasing attention is being given to enhancing the overall quality of life of older people and enabling them to lead an active, healthy, and independent lives. A key aim is to support older people to continue living in their own homes and communities, and cater to their needs by optimizing the provision of health services and other forms of support.

This more holistic and personalized approach may be supported by a range of emerging technologies. For example, the use of surveillance and AI-based technologies to collect and process large amounts of data allows intelligent systems to create safer and more supportive living environments (Sixsmith, 2013). AI-based assistive technologies may integrate data from sensors and monitoring devices, such as smart floors or smart beds, with data from smart wearable sensors and smart home tools that measure the gas, water and power consumption of users (Krick et al., 2019; Rubeis, 2020. These systems may enhance the sharing of information between users and caregivers and between different sectors to create a more integrated care system.). Assistive robots may assist older people in their daily activities, support them in maintaining mental and physical health, and provide psychosocial support (García-Soler & Díaz-Orueta 2018; Piloto et al., 2018).

Although AgeTech can be seen as an opportunity for empowering older people and enhance their overall quality of life, crucial ethical issues have to be addressed. By changing the ways of interaction and communication between health service providers and older people, AgeTech transforms the practices involved and the relationships central to care provision. In addition, different technologies come with a bundle of ethical issues and potential risks, from data security and privacy protection to algorithmic bias and digital ageism. Hence, ethical issues in AgeTech are multifaceted, arise in different contexts, and are in part determined by the cross-sectoral organization of health services. This begs the question, how can core values of providing health services, such as humanity, reciprocity, and empathy, be integrated into the development and implementation of AgeTech?

It is also important to discuss the concepts and assumptions about aging, health and quality of life that may underpin tech development. Given the fact that AgeTech is often proposed as a solution for overcoming the twin issue of the population aging and the demands on health professionals and family caregivers, how can we make sure that digital solutions are implemented for the benefit of end users and not just for reasons of efficiency and cost-effectiveness? What ethical aspects are connected to the human-machine interaction, especially in robotics? How can the autonomy of end users be secured when techniques of monitoring and surveillance are implemented, for example in the context of Ambient Assisted Living (AAL) systems and telenursing? How can prevent social scripts like gender and age stereotypes from being inscribed into the technology?

The articles in this topical collection focus on these and other ethical questions, particularly in respect to key emerging technologies of AI and robotics. The overall aim is to explore the multifaceted ethical landscape of emerging AgeTech and to provide frameworks and strategies for ethically-appropriate technologies that support the health, well-being, and quality of life of older adults.

**Simona Tribelli** investigates Health Recommender Systems (HRS) as AI-based tools for enabling healthy lifestyles and therapy adherence. One crucial benefit of HRS is that they could be tools for supporting healthy and active aging. However, the ethical aspects linked to HRS are yet poorly understood and articulated. Tribelli explores the ethical aspects by focusing on autonomy in the context of HRS for active ageing. She outlines the technical aspects and the associated risks and introduces a categorization of ethical challenges in order to better understand and possibly prevent or mitigate these risks. Based on the principle of autonomy, the paper proposes an autonomy-based ethical framework for the development HRS that enables active ageing.

The paper by **Merle Weßel**,** Niklas Ellerich‑Groppe**,** Frauke Koppelin**, and **Mark Schweda** focuses on gender and age stereotypes in robotics used in care for older people. This technology relies mostly on social categorizations of gender or age, which shape the interaction between humans and robots. The authors found that that these stereotypical categorizations are even advocated as means to improve acceptance, well-being, and quality of life of older people. From an ethical point of view, this raises questions of autonomy and discrimination against certain users. The article investigates how tech developers and nursing practitioners perceive and evaluate social categorizations of gender and age in robotics. The authors follow an empirical approach, using semi-structured stakeholder interviews to explore the awareness, evaluations, and lines of argument regarding the ethical issues at hand. Their findings suggest that there are six different approaches for dealing with gender and age stereotypes in this context: negation, functionalistic relativization, explanation, neutralization, stereotyping, and queering. The authors emphasise the principle of professional responsibility for the creation and implementation of ethically-appropriate AgeTech in pluralistic societies.

**Giovanni Rubeis**,** Mei Lan Fang**, and **Andrew Sixsmith** focus on the principle of equity in regard to AgeTech for ageing well. The authors explore the role of social determinants in designing AI-based AgeTech. These technologies are mainly designed to support the health and independent living of older people, provide social connectedness, support wellbeing and mental health, and enable social participation. The authors investigate the ethical aspects involved through a critical analysis of ethical design, digital equity, and policy pathways. Their basic assumption is that equity is key for AI-based AgeTech in order to realize its full potential in terms of driving practical, equitable, and inclusive multilevel solutions to support healthy, active ageing. The authors conclude that equity is not a just a mere add-on, but should be the crucial aim of designing AI-based AgeTech. One important task in this regard is to address social determinants that affect the use of or access to these technologies. Another crucial factor is complexity management, which is a technical necessity in AI systems, but may also create and exacerbate social inequities by marginalizing or ignoring social factors. The critical analysis focused on equity shows that bias, standardization, and access are the main ethical issues connected to AI-based AgeTech. Based on their results, the authors make recommendations for tackling inequities in AI-based AgeTech.

In their article, **Joschka Haltaufderheide**,** Annika Lucht**,** Christoph Strünck** and **Jochen Vollmann** provide a systematic review of the empirical evidence on socially assistive devices in healthcare. While robot caregivers or companions are seen as potentially important technologies for improving healthcare services, they present various ethical issues. The systematic review focuses on findings from non-comparative empirical studies and investigates the interaction with these devices, how it shapes the perceptions of users, as well as their ability to express themselves from an ethical perspective. While many users have a positive attitude and emotional reaction towards socially assistive devices, the perceptions of social relations vary across users. Due to a lack of understanding of technical complexities on behalf of users and limited functionality of the devices, behavioral alignment of users is often observed as a prerequisite for making use of the devices. Hence, three ethical aspects are crucial in this context, the reliability of existing empirical evidence to inform normative judgements, the ethical significance of the social presence of devices, and user autonomy in regard to behavioral alignment.
